# Case Report: Longitudinal temporomandibular joint remodeling in MMP2 associated nodulosis arthropathy osteolysis syndrome during serial panoramic follow up

**DOI:** 10.3389/fdmed.2026.1876677

**Published:** 2026-07-15

**Authors:** Abdullah Alzahem, Abdullah Alghamdi, Wael Aboelmaaty

**Affiliations:** 1 Department of Medical Education, College of Medicine, King Saud bin Abdulaziz University for Health Sciences (KSAU-HS), Riyadh, Saudi Arabia; 2 Dental Department, Ministry of National Guard Health Affairs (MNGHA), Riyadh, Saudi Arabia; 3 King Abdullah International Medical Research Center (KAIMRC), Riyadh, Saudi Arabia; 4 Maxillofacial Surgery and Diagnostic Sciences Department, College of Dentistry, King Saud bin Abdulaziz University for Health Sciences, Riyadh, Saudi Arabia; 5 Oral Radiology and Diagnostic Sciences Department, Faculty of Dentistry, Mansoura University, Mansoura, Egypt

**Keywords:** case report, MMP2, multicentric osteolysis, nodulosis, and arthropathy, nodulosis-arthropathy-osteolysis syndrome, panoramic radiography, temporomandibular joint

## Abstract

Nodulosis-arthropathy-osteolysis (NAO) syndrome, within the multicentric osteolysis, nodulosis, and arthropathy (MONA) spectrum, is a rare autosomal recessive osteolytic disorder most commonly associated with pathogenic variants in MMP2. Appendicular skeletal involvement is well recognized, whereas temporomandibular joint (TMJ) involvement remains poorly documented. We report longitudinal clinical and radiographic TMJ findings in an adult male with genetically confirmed MMP2-associated NAO syndrome, with the exact variant to be reported using HGVS nomenclature from the genetic report who underwent serial panoramic radiographic follow-up between 2018 and 2026. Over the observation period, panoramic images demonstrated progressive bilateral condylar flattening, cortical irregularity, lateral osteophytic change, shallowing of the glenoid fossae, and marked flattening of the articular eminences. Despite progressive structural remodeling, symptoms remained relatively mild, with intermittent TMJ clicking, mild discomfort, and generally preserved mandibular function. The case is further notable because the patient received long-term intravenous antiresorptive therapy, first with pamidronate and later with zoledronic acid, requiring explicit consideration of medication-related osteonecrosis of the jaw (MRONJ). No documented clinical evidence of MRONJ was identified during follow-up. This case suggests that TMJ remodeling may be part of the craniofacial phenotype of NAO/MONA spectrum disease and illustrates both the value and limitations of serial panoramic radiography. Panoramic imaging can document gross longitudinal osseous change, but the absence of CBCT and MRI limits three-dimensional bony and soft-tissue assessment.

## 1 Introduction

Nodulosis-arthropathy-osteolysis (NAO) syndrome is part of the multicentric osteolysis, nodulosis, and arthropathy (MONA) spectrum, an ultra-rare inherited skeletal disorder caused most commonly by pathogenic variants in MMP2, which encodes matrix metalloproteinase-2 (MMP-2) (1–4). The disorder is inherited in an autosomal recessive pattern and usually begins in childhood with progressive osteolysis affecting the hands and feet, arthropathy, reduced mobility, generalized osteopenia or osteoporosis, and variable connective-tissue manifestations such as subcutaneous nodules, skin changes, gingival overgrowth, and ocular findings (1, 4–7).

Although appendicular skeletal manifestations are well described, craniofacial and temporomandibular joint (TMJ) involvement remains insufficiently characterized. This gap is clinically important because the TMJ is a load-bearing synovial joint exposed to repeated functional stresses during mastication, speech, and mandibular movement. In a patient with disturbed extracellular-matrix turnover and systemic osteolysis, the TMJ may be vulnerable to progressive remodeling even when symptoms are mild.

**Figure 1:**
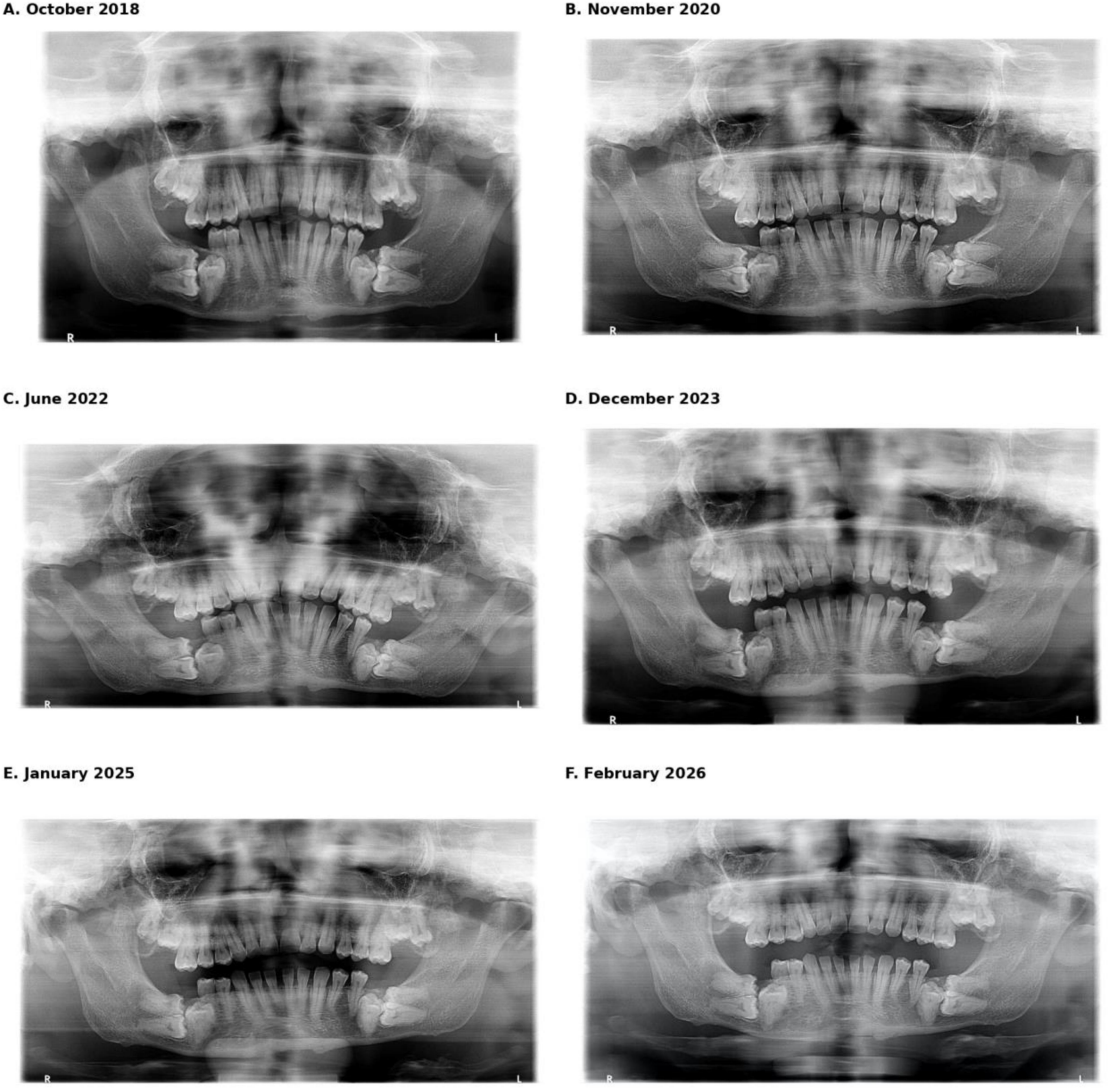
Serial panoramic radiographs obtained between 2018 and 2026 showing progressive bilateral temporomandibular joint remodeling in an adult patient with genetically confirmed NAO syndrome. **(A)** October 2018: bilateral flattening of the superior condylar contours and articular eminences with shallow glenoid fossae; cortical outlines remain continuous. **(B)** November 2020: persistent condylar flattening with mild lateral osteophytic change, more conspicuous on one condyle, and greater flattening of the articular eminences. **(C)** June 2022: further bilateral flattening of the condylar surfaces with increased apparent shallowing of the glenoid fossae. **(D)** December 2023: no major interval change relative to 2022. **(E)** January 2025 and **(F)** February 2026: advanced remodeling with irregular but continuous cortical outlines of the condyles and marked flattening of the articular eminences with loss of normal posterior slope. Interpretation is limited by inherent magnification, distortion, and superimposition of panoramic imaging and by incomplete visualization of the left TMJ in several panels. Only one unlabelled set of radiographs **(A–F)** is provided.

Imaging is central to assessment of osseous TMJ abnormalities. Panoramic radiography is accessible and useful for longitudinal monitoring of gross morphology, but it is limited by magnification, distortion, projection variability, and superimposition. Cone-beam computed tomography (CBCT) provides more detailed three-dimensional assessment of cortical and trabecular bony changes, whereas magnetic resonance imaging (MRI) is superior for evaluating articular disc position, effusion, marrow signal, and soft-tissue/internal derangement (8–12). We present an eight-year panoramic follow-up of TMJ remodeling in genetically confirmed NAO syndrome, with particular attention to differential interpretation, long-term antiresorptive therapy, MRONJ surveillance, and limitations imposed by the absence of CBCT and MRI. The longitudinal panoramic radiographic findings are shown in Figure 1.

## 2 Case description

### 2.1 Patient information

The patient was a male born in 1994 to consanguineous parents. He initially presented during childhood with progressive swelling and stiffness affecting the small joints of the hands and feet, accompanied by subcutaneous nodules. Genetic testing confirmed NAO syndrome associated with a pathogenic MMP2 variant. Exact HGVS nomenclature, including reference transcript, coding DNA change, protein consequence, and zygosity, was not available in the retrospective clinical record. The patient remained under multidisciplinary medical care for systemic skeletal manifestations of the disorder.

At the time of oral and maxillofacial evaluation, the patient reported no history of maxillofacial trauma or previous TMJ surgery. He was followed clinically and radiographically as part of routine dental and craniofacial monitoring. No CBCT or MRI examinations were performed during the follow-up period. The available clinical records did not document MRONJ-related findings, exposed jaw bone, probeable fistula, or other clinical evidence of medication-related osteonecrosis of the jaw.

### 2.2 Clinical findings

Clinical evaluation included patient-reported symptoms and objective examination of the masticatory system. The patient reported intermittent jaw joint sounds, mild discomfort during mandibular movement, headache, and bruxism. There was no reported jaw locking or severe restriction in mouth opening. On examination, bilateral TMJ tenderness and clicking were present. Tenderness of the temporalis muscles was also noted bilaterally, while the sternocleidomastoid muscles were non-tender and the masseter muscles showed increased tension without tenderness.

Mandibular function was generally preserved. No deviation or deflection was observed during mandibular opening, and lateral mandibular movements were not associated with pain. Intraoral examination did not reveal occlusal wear or cheek-bite keratosis. The latest available mouth-opening values were: maximum comfortable opening 36 mm, maximum unassisted opening 37 mm, and maximum assisted opening 38 mm. The longitudinal records did not contain complete standardized DC/TMD variables for every imaging year, which is acknowledged as a limitation of this retrospective case review.

### 2.3 Timeline

Table 1 summarizes the available longitudinal clinical, imaging, and therapeutic information.

**Table 1:** Longitudinal clinical, radiographic, and therapeutic timeline of the patient with MMP2-associated nodulosis-arthropathy-osteolysis syndrome.

Time point	Radiographic findings	Clinical findings	Antiresorptive therapy/care context	Notes
Oct 2018	Bilateral condylar flattening; shallow glenoid fossae; flattening of articular eminences; cortical outlines continuous.	Full standardized TMJ clinical metrics not available for this time point.	Ongoing cyclical IV pamidronate therapy.	Baseline panoramic evidence of established TMJ remodeling.
Nov 2020	Persistent flattening; mild lateral osteophytic change, more conspicuous on one side; further articular eminence flattening.	Intermittent TMJ sounds reported during follow-up overall.	Yearly IV zoledronic acid had begun after treatment transition in 2019.	Left TMJ less clearly visualized than right in available panoramic image.
Jun 2022	Severe bilateral condylar flattening; increased apparent shallowing of glenoid fossae.	Mild symptoms overall; no severe functional limitation recorded.	Yearly IV zoledronic acid ongoing.	Progression visible on serial panoramic comparison.
Dec 2023	No major interval change compared with 2022.	Longitudinal symptom data incompletely standardized in the retrospective chart.	Yearly IV zoledronic acid ongoing.	Limited granularity of clinical follow-up records.
Jan 2025	Advanced remodeling with irregular but continuous cortical outlines and severe flattening of articular eminences.	Intermittent clicking; mandibular function generally preserved.	Yearly IV zoledronic acid ongoing.	No CBCT/MRI available.
Feb 2026	Persistent advanced remodeling with marked loss of normal articular eminence contour and posterior slope.	Mild discomfort and intermittent clicking; latest opening values within functional range.	Yearly IV zoledronic acid ongoing.	No documented clinical evidence of MRONJ during follow-up.

### 2.4 Diagnostic assessment

Serial panoramic radiographs obtained between October 2018 and February 2026 were retrospectively reviewed to assess longitudinal structural changes affecting both TMJs. The descriptive assessment focused on condylar morphology, cortical continuity and irregularity, osteophyte formation, glenoid fossa depth, and articular eminence contour. A standardized TMJ scoring system was not applied because no validated scoring framework exists specifically for NAO/MONA-associated TMJ osteolysis on serial panoramic radiographs. In addition, most standardized TMJ imaging criteria were developed for degenerative TMJ disease and/or require CBCT or MRI for reliable assessment of three-dimensional osseous or soft-tissue features, which were not available in this case. Therefore, a structured descriptive framework based on reproducible panoramic morphological features was used to support transparent longitudinal comparison without implying a level of diagnostic precision that the available imaging modality could not provide.

Two experienced oral and maxillofacial radiologists independently reviewed the serial radiographs and reached a consensus interpretation. The images were reviewed under routine digital diagnostic viewing conditions available to the treating center. Exact software version, monitor calibration, and ambient-light details were not retrievable from the retrospective record. Complete chronological blinding was not feasible because the objective was longitudinal comparison of serial radiographs and the images contained acquisition dates and age-related features.

The principal diagnostic challenge was distinguishing NAO-associated TMJ osteolysis from other causes of condylar remodeling. The differential interpretation included degenerative TMJ disease/osteoarthritis, adaptive remodeling related to functional loading or bruxism, post-traumatic remodeling, inflammatory arthropathy, and medication-related jaw complications. Prior trauma and TMJ surgery were not reported. Bruxism and muscle tenderness may have modified joint loading, but the bilateral, progressive pattern in a patient with genetically confirmed systemic osteolysis supports NAO/MONA-associated craniofacial involvement as the dominant explanatory context. MRONJ was considered because of prolonged intravenous bisphosphonate exposure, but no clinical evidence of exposed bone, probeable fistula, or jaw necrosis was documented.

### 2.5 Therapeutic intervention

The patient was managed in a multidisciplinary setting for progressive systemic osteolysis. The patient received cyclical intravenous pamidronate therapy from 2010 to 2019, administered over 3 consecutive days every 3 months. In 2019, treatment was transitioned to intravenous zoledronic acid, which has been administered as a single 5 mg infusion once yearly thereafter.

The purpose of antiresorptive therapy was to reduce osteoclastic bone resorption and help slow the progression of osteolytic skeletal disease. No TMJ-specific surgical intervention was performed. Oral and maxillofacial care consisted of clinical monitoring, symptom assessment, and periodic panoramic radiographic follow-up. The available records did not document antiresorptive-related jaw adverse events or clinical MRONJ during the reviewed period.

### 2.6 Follow-up and outcomes

At the initial panoramic evaluation in 2018, both mandibular condylar heads showed flattening, shallow glenoid fossae, and flattening of the articular eminences, while the cortical outlines remained continuous. Follow-up imaging in 2020 demonstrated persistent bilateral condylar flattening and mild lateral osteophytic change, more conspicuous on one condyle, together with greater flattening of the articular eminences. In 2022, further bilateral flattening and increased apparent shallowing of the glenoid fossae were observed. The 2023 image showed no major interval change compared with 2022.

By 2025 and 2026, the panoramic radiographs demonstrated advanced remodeling, including irregular but continuous condylar cortical outlines and marked flattening of the articular eminences with loss of normal posterior slope. These findings indicate chronic progressive structural remodeling affecting both the mandibular condylar and temporal components of the TMJ. However, visualization of the TMJ regions, especially the left TMJ in several panels, was incomplete, limiting confidence for subtle interval changes.

Clinically, the course demonstrated a relative dissociation between radiographic progression and symptom severity. The patient continued to report intermittent TMJ clicking and mild discomfort, but mandibular function remained generally preserved and mouth opening stayed within functional limits. Follow-up was therefore continued to monitor structural progression, clinical symptoms, and potential oral complications related to long-term antiresorptive therapy.

## 3 Discussion

This case report provides longitudinal evidence that the TMJ may be involved in the craniofacial phenotype of MMP2-associated NAO/MONA spectrum disease. The eight-year panoramic sequence demonstrates progressive bilateral remodeling of the condyles, glenoid fossae, and articular eminences, while the clinical course remained comparatively mild. This radiographic-clinical dissociation is important because structural degenerative changes in the TMJ do not always correlate closely with pain severity or functional limitation (13, 14).

The observed pattern is biologically plausible in the setting of MMP-2 dysfunction. MMP-2 contributes to extracellular-matrix turnover, collagen remodeling, and skeletal homeostasis. Loss or impairment of MMP-2 activity can disturb the balance between matrix degradation, bone resorption, and repair, producing progressive osteolysis, joint destruction, and deforming arthropathy (2–7). Within the TMJ, repetitive functional loading may interact with abnormal bone and connective-tissue metabolism, promoting flattening, cortical irregularity, and adaptive remodeling over time.

At the same time, the imaging findings are not pathognomonic for NAO. Condylar flattening, cortical irregularity, and osteophytic change can also be seen in degenerative TMJ disease and may be influenced by parafunctional loading. The patient reported bruxism and muscle findings compatible with altered masticatory loading; however, no occlusal wear or cheek-bite keratosis was identified, and the bilateral progressive changes occurred in the context of genetically confirmed systemic osteolysis. The most balanced interpretation is therefore that NAO/MONA-associated osteolytic arthropathy likely provided the systemic substrate for TMJ remodeling, while local biomechanical factors may have acted as modifiers rather than sole causes.

Long-term antiresorptive therapy is a key contextual feature. Published experience in MONA spectrum disorders suggests that bisphosphonates such as pamidronate or zoledronate may reduce skeletal pain and improve bone mineral density, but established deformity and restricted range of motion may persist (15, 16). Therefore, continued TMJ remodeling during treatment should not be interpreted as direct evidence of treatment failure or drug causality. In this case, pamidronate followed by zoledronic acid was used as part of multidisciplinary management of progressive systemic osteolysis, and the available data do not allow causal attribution of TMJ progression to antiresorptive therapy.

Nevertheless, prolonged intravenous bisphosphonate exposure required explicit MRONJ consideration. The AAOMS position paper defines MRONJ by clinical criteria that include current or previous antiresorptive or antiangiogenic therapy, exposed bone or bone that can be probed through a fistula in the maxillofacial region persisting for more than 8 weeks, and no history of jaw radiation therapy or metastatic jaw disease (17). In this patient, the available follow-up records did not document exposed bone, probeable fistula, non-healing jaw necrosis, or other clinical evidence of MRONJ. The absence of MRONJ findings should be reported cautiously as a clinical observation rather than proof of absence of risk, because cumulative antiresorptive exposure still justifies careful oral surveillance, avoidance of unnecessary invasive dentoalveolar procedures when possible, and prompt evaluation of any non-healing oral lesion.

The major methodological limitation is exclusive reliance on panoramic radiography. Serial panoramic imaging was useful because it provided an accessible, low-burden method for documenting gross osseous TMJ change over several years. However, panoramic images are affected by magnification, distortion, superimposition, and positioning variation, and the TMJ region may be incompletely delineated. These limitations are visible in the present figure set, particularly for the left TMJ in several panels. The absence of CBCT limits three-dimensional assessment of cortical erosion, osteophyte morphology, subcortical change, and fossa/eminence remodeling (8, 10–12). The absence of MRI prevents assessment of disc position, joint effusion, marrow signal, retrodiscal tissues, and internal derangement, which is particularly relevant because intermittent TMJ clicking can be associated with disc displacement or functional joint disturbance (9, 14).

Additional limitations include the single-patient design, retrospective data collection, incomplete standardized longitudinal DC/TMD documentation, and limited image-review metadata. Exact viewing software, monitor calibration, and ambient-light conditions were not retrievable. Complete chronological blinding was not feasible because the study objective was longitudinal comparison. These limitations restrict generalizability and prevent precise quantification of progression. However, the case remains valuable because long-term adult TMJ follow-up in genetically confirmed NAO syndrome is rarely documented, and the combination of serial imaging, clinical follow-up, and antiresorptive therapy context provides practical information for oral and maxillofacial clinicians managing patients with systemic osteolytic disorders.

## 4 Conclusion

This case suggests that progressive TMJ remodeling may occur as part of the craniofacial phenotype of MMP2-associated NAO/MONA spectrum disease. Serial panoramic radiographs documented major longitudinal osseous change over eight years, while symptoms remained mild and mandibular function was preserved. The findings should be interpreted cautiously because CBCT and MRI were not performed, and panoramic imaging cannot fully characterize three-dimensional bony changes or soft-tissue/internal derangement. In patients with rare systemic osteolytic disorders, recognition of possible TMJ involvement may support earlier multidisciplinary assessment, ongoing oral surveillance, and more deliberate imaging selection when clinical symptoms or radiographic progression warrant further evaluation.

## 5 Patient perspective

The patient reported intermittent jaw discomfort and joint sounds during mouth opening and chewing over several years. Although the symptoms were generally mild, they occasionally caused concern during daily activities such as eating and speaking. The patient said the jaw sounds and mild discomfort worried him during eating and speaking, but he was reassured by regular follow up. He also felt reassured that the findings were being carefully documented and followed over time to improve understanding of the progression of his condition.

## Data Availability

The original contributions presented in the study are included in the article/Supplementary Material, further inquiries can be directed to the corresponding author.
